# Modeling Duchenne Muscular Dystrophy Cardiomyopathy with Patients’ Induced Pluripotent Stem-Cell-Derived Cardiomyocytes

**DOI:** 10.3390/ijms24108657

**Published:** 2023-05-12

**Authors:** Binyamin Eisen, Ofer Binah

**Affiliations:** Cardiac Research Laboratory, Department of Physiology, Biophysics and Systems Biology, Rappaport Faculty of Medicine and Research Institute, Technion—Israel Institute of Technology, Haifa 3200003, Israel

**Keywords:** Duchenne muscular dystrophy, DMD, *dystrophin* gene, dystrophin protein, human induced pluripotent stem-cell-derived cardiomyocytes, hiPSC-CMs, dilated cardiomyopathy, DCM, arrhythmias, depressed cardiac function

## Abstract

Duchenne muscular dystrophy (DMD) is an X-linked progressive muscle degenerative disease caused by mutations in the *dystrophin* gene, resulting in death by the end of the third decade of life at the latest. A key aspect of the DMD clinical phenotype is dilated cardiomyopathy, affecting virtually all patients by the end of the second decade of life. Furthermore, despite respiratory complications still being the leading cause of death, with advancements in medical care in recent years, cardiac involvement has become an increasing cause of mortality. Over the years, extensive research has been conducted using different DMD animal models, including the *mdx* mouse. While these models present certain important similarities to human DMD patients, they also have some differences which pose a challenge to researchers. The development of somatic cell reprograming technology has enabled generation of human induced pluripotent stem cells (hiPSCs) which can be differentiated into different cell types. This technology provides a potentially endless pool of human cells for research. Furthermore, hiPSCs can be generated from patients, thus providing patient-specific cells and enabling research tailored to different mutations. DMD cardiac involvement has been shown in animal models to include changes in gene expression of different proteins, abnormal cellular Ca^2+^ handling, and other aberrations. To gain a better understanding of the disease mechanisms, it is imperative to validate these findings in human cells. Furthermore, with the recent advancements in gene-editing technology, hiPSCs provide a valuable platform for research and development of new therapies including the possibility of regenerative medicine. In this article, we review the DMD cardiac-related research performed so far using human hiPSCs-derived cardiomyocytes (hiPSC-CMs) carrying DMD mutations.

## 1. Duchenne Muscular Dystrophy

Duchenne muscular dystrophy (DMD) is an X-linked progressive muscle degenerative disease caused by mutations in the *dystrophin* gene with an estimated prevalence between 1.3 and 2.1 per 10,000 live male births [1,2,3]. *Dystrophin* is the longest gene in the human DNA spanning 2.4 Mbp, with its 14 kb transcript consisting of 79 exons; it encodes the 427 kDa dystrophin protein [4,5]. Dystrophin is a major structural protein which is also involved in important metabolic processes [6,7,8]. In skeletal and cardiac muscle, dystrophin provides mechanical stability essential for contracting myocytes, and anchors the cellular cytoskeleton to the extracellular matrix (ECM) via the transmembrane dystrophin–glycoprotein complex (DGC) which links directly to extracellular laminin-2 [9,10,11]. Dystrophin can be grossly divided into three major domains: (1) the N-terminus and actin-binding domain (ABD) (exons 1–8), (2) the central rod segment consisting of 24 spectrin repeats (exons 9–63), and (3) the DGC-binding domain and C-terminus (exons 64–79) [12,13]. The majority of DMD mutations are deletions of one or more exons (60–65%), while duplications make up to 5–10% of the cases. The remaining (25–35%) are single-nucleotide variants, small deletions or insertions in the coding sequence, or splice site variants [14,15].

DMD symptoms start at an early age, usually around 2–3 years, when proximal muscles of the lower extremities begin to weaken. Gradually, the weakness progresses to the distal muscles and the upper limbs. With age, symptoms become more prominent, and by the early teens, patients are usually wheelchair-dependent. The cardiac involvement of DMD includes dilated cardiomyopathy (DCM) which is present in virtually all patients by their late teen years, along with conduction abnormalities, various arrhythmias and extensive fibrosis. Eventually, patients die by their late 20s or early 30s due to respiratory and cardiac failure [16,17,18].

The current gold-standard treatment of DMD includes glucocorticoids (GCs), usually from the age of 4 years, aimed at improving motor and pulmonary function, while also potentially delaying the onset of DCM [16,19,20]. Beyond known side-effects including weight gain, hirsutism and other Cushing’s syndrome symptoms, GCs do not change the disease outcome, but rather can only slow its course [19,21]. Angiotensin-converting enzyme (ACE) inhibitors and angiotensin receptor blockers (ARBs) are administered from around the age of 10 years to reduce mechanical stress on the heart [16,17]. Novel therapies include Eteplirsen, an exon 51 skipping drug, and Ataluren (PTC124), which promotes ribosomal readthrough of nonsense mutations [22,23]. However, these treatments are not intended for all DMD mutations, and there is a need for additional research and therapies to be developed.

The primary animal model used for DMD research is the *mdx* mouse, which carries a nonsense point mutation in exon 23 of the *dystrophin* gene [24,25]. Although *mdx* mice exhibit chronic degeneration of myofibers, they do not manifest some prominent symptoms of DMD. *mdx* mice display a slower disease progression compared to human DMD patients, and their relative lifespan is significantly longer. The slow progression of muscle pathology does not lead to extensive fibrosis as in humans, and the mice retain their mobility. Cardiac involvement follows a different course than in humans, as *mdx* mice initially develop hypertrophic cardiomyopathy (HCM), while human DMD patients suffer from contractile dysfunction and DCM [26,27,28]. It has been previously found that *mdx* mice heart mitochondria display an increase in Ca^2+^ uptake rate via activation of Ca^2+^ transport, possibly compensating for a defective sarcoplasmic reticulum (SR) [29,30,31]. This adaptiveness of *mdx* mice may be a key feature differentiating this model from human patients [32]. Indeed, these challenges led to the development of the D2.*mdx* model which exhibits a significantly more prominent disease phenotype [33]. An additional limitation of the mouse model lies in gender differences between female and male *mdx* mice. These differences include a more prominent cardiac involvement and skeletal muscle degeneration in female compared to male mice [34,35], contrary to slower disease progression in human female carriers [36].

Another important animal model was developed in rats by means of TALENs targeting *DMD* exon 23 [37]. *mdx* rats manifest progressive muscle degeneration accompanied by a reduction in muscle force, as well as dilated cardiomyopathy. Importantly, *mdx* rats display significant fibrosis in skeletal and cardiac muscle, similar to human patients but contrary to *mdx* mice [37,38]. However, some differences remain between *mdx* rats and human patients, as well as cells derived from human patients, including the lack of muscle calcifications [37], normal L-type Ca^2+^ current (I_Ca,L_) [39,40,41], and unimpaired β-adrenergic cascade [39,42,43,44] in *mdx* rats.

## 2. Human Induced Pluripotent Stem Cells

In 2006 Takahashi and Yamanaka published their successful attempt to reprogram differentiated rat somatic cells into induced pluripotent stem cells (iPSCs) by means of the induction of four factors: Oct3/4, SOX2, c-Myc, and KLF4 [45]; in 2007, these breakthroughs were repeated in human somatic cells [46]. Human iPSCs (hiPSCs) present classic embryonic stem-cell (ESC) characteristics including trilineage differentiation capability [47,48]. Thus, provided proper culture and media conditions, hiPSCs can be differentiated into various cell types. Like ESCs, hiPSCs and hiPSC-derived cells can be used for disease modeling and drug testing [49,50,51,52,53]. Furthermore, hiPSCs can generate a potentially endless pool of differentiated cells from a minute biopsy of a single living human donor, whereas ESC generation requires the sacrifices of embryos [54]. This enables previously unmatched research capabilities of various human diseases without the limitations of different animal models. Indeed, in the past years, numerous papers utilized the reprogramming technique for disease modeling and regenerative medicine. Patient-specific hiPSCs served as a means for many discoveries and advancements in research of different diseases [51,52,55,56,57].

Due to the multitude of different mutations causing DMD [58], patient-specific hiPSCs provide a valuable approach to investigate the precise disease mechanisms resulting from these mutations. Furthermore, hiPSC research enables the potential development of new drugs and therapeutic approaches targeting specific mutations with higher efficacy than previous generic treatments. Importantly, investigating human-derived cells is preferable to using animal models which display different disease course and characteristics, compared to human patients.

## 3. Gene Expression Changes in DMD hiPSC-CMs

DMD has been previously linked to changes in cellular gene expression in different models including *mdx* mice, as well as in human patients [59,60,61,62]. To support hiPSCs as a valid model for DMD modeling and gain better understanding of the disease mechanisms, it is also imperative to investigate gene expression changes in DMD hiPSC-CMs. Lin et al. [41] discovered that DMD hiPSC-CMs exhibit higher rates of cell death compared to healthy cells, demonstrated by increased staining for CASP3 and elevated levels of DNA fragmentation. Additionally, they found extensive changes in gene expression including important apoptosis regulators such as *CASP3*, *CASP8*, *CASP9* and antiapoptotic *XIAP,* [63], genes involved in contractility such as *MYL2*, *MYL3*, *ACTN1* and *TPM1* [64], and genes associated with heart diseases such as *MAPK11*, *COL3A1* and *CALM1* [65,66,67]. Moreover, bio-functional enrichment analysis of the genes revealed that categories related to heart disease conditions were positively enriched, while others involved in muscle development and contractility were negatively enriched in DMD hiPSC-CMs. Importantly, these enrichment patterns are consistent with clinical observations in DMD patients [68,69,70,71]. By means of whole-transcriptome sequencing analysis, Lin and coworkers discovered that alongside elevated expression of *CASP3* and *DIABLO*, *XIAP* was decreased in DMD hiPSC-CMs, indicating that possible mitochondrial involvement in DMD hiPSC-CMs increased apoptosis [63]. Accordingly, transmission electron microscopy (TEM) demonstrated swollen mitochondria in DMD hiPSC-CMs [41]. Furthermore, FACS analysis of DMD hiPSC-CMs using JC-1 mitochondrial membrane potential staining revealed an increase in damaged mitochondria in DMD hiPSC-CMs [41]. Overall, Lin et al.’s findings suggest that a common mitochondria-mediated signaling network is involved in elevated apoptosis in DMD hiPSC-CMs.

Chang and coauthors [72] investigated the involvement of telomere shortening in various cardiomyopathies including DMD. Quantitative fluorescence in situ hybridization (Q-FISH) and quantitative polymerase chain reaction (qPCR) demonstrated increased telomere shortening in DMD compared to healthy hiPSC-CMs. The telomere has been shown be involved in gene expression of endothelial cells, and overexpression of telomerase protein (TERT) in mouse cardiomyocytes was proven to protect from myocardial ischemia [73,74,75]. Interestingly, *mdx* mice, which do not express cardiac symptoms as extensive as human DMD patients, have substantially longer telomeres than human patients [76]. Indeed, genetically engineered *mdx* mice with shortened telomeres developed severe heart failure similar to humans. The same group later discovered [77] by means of qRT-PCR, decreased transcript levels of telomere repeat-binding proteins (TRF1 and TRF2) and shelterin complex proteins (RAP1, TIN2, and POT1) in DMD compared to healthy cardiomyocytes, indicating possible involvement of these absent proteins in telomere shortening. Additionally, this group also found upregulation of p53 and p53-binding protein 1 (53BP1), indicating activation of the DNA damage response, as also demonstrated by increased apoptotic markers caspase-3, cleaved PARP, and accumulation of β-galactosidase signal in DMD hiPSC-CMs compared to healthy cells. Lastly, blocking the contraction of cardiomyocytes with blebbistatin, which locks the myosin heads in a low-affinity state preventing actin binding [78], abolished telomere shortening. These findings highlight aberrant contraction as an important factor involved in telomere shortening and shelterin downregulation, eliciting a p53-dependent DNA damage response.

Farini et al. [79] investigated the involvement of immunoproteasome (IP) dysregulation in the cellular pathophysiology of DMD. The group reported an increase in the expression of IP subunits PSMB8 and PSMB9 in DMD compared to healthy cardiomyocytes. Accordingly, administration of ONX-0914, an IP inhibitor, decreased intracellular Ca^2+^ levels, as well as the release of cTnl, implying increased cell survival. Additionally, IP inhibition resulted in downregulation of TGF-β and type III collagen-α, suggesting reduced fibrosis [80]. Overall, these results demonstrate IP as a potential pharmacological target for DMD patients and its involvement in cardiac pathophysiology.

Our group [40] reported expression changes in genes encoding ion channels in DMD hiPSC-CMs, including *HCN* which encodes the channel responsible for the pacemaker funny current (I_f_) during phase 4 of the cardiac action potential, as well as reduced I_f_ density compared to healthy cells. Additionally, we found in DMD cardiomyocytes, increased expression of the *CACNA1C* gene, which encodes the channel responsible for the I_Ca,L_ current, and we discovered that I_Ca,L_ density increased accordingly.

Kamdar and colleagues [44] reported increased expression of known fibrosis genes *COL1A1, ADAMTS2, COL6A1* and *THY1* in DMD cardiomyocytes compared to healthy cells, consistent with other reports [79]. Additionally, Kyoto Encyclopedia of Genes and Genomes (KEGG) pathway analysis of dysregulated genes found that DMD hiPSC-CMs demonstrated upregulation of genes associated with extracellular matrix (ECM) organization, cellular proliferation and fibrosis, as well as downregulation of genes associated with intracellular Ca^2+^ handing and contraction.

Jelinkova et al. [42] found an increase in K^+^ channel Kir2.1 expression, despite no difference in action potential parameters between DMD and healthy hiPSC-CMs, contrary to other studies [40]. Furthermore, immunostaining demonstrated increased levels of the I_Ca,L_ channel expression in DMD cardiomyocytes. However, contrary to our report [40], at the mRNA level there was no difference, indicating increased cellular localization rather than differences in transcription.

Our group also reported [43] changes in gene expression patterns in DMD hiPSC-CMs. RNA-seq analysis revealed 119 genes included in KEGG pathways for cardiac intracellular Ca^2+^ handling, contraction and adrenergic signaling. Western blot (WB) analysis of SERCA2 demonstrated overexpression in DMD compared to healthy cells, possibly a compensatory attempt for impaired intracellular Ca^2+^ handling. Furthermore, downregulation of the β1 adrenergic receptor (ADRβ1) and adenylate cyclase (AC) demonstrated by RNA-seq, combined with depleted SR Ca^2+^ stores, likely underlies the blunted β-adrenergic positive inotropic response of DMD cells.

Yasutake et al. [81] investigated the involvement of the yes-associated protein (YAP, an important transcriptional factor involved in cell proliferation, growth and regeneration) in DMD cardiomyopathy. Previous research found decreased YAP activity in dystrophic muscles, resulting in poor regeneration and damage [82]. Interestingly, in DMD cardiomyocytes, the YAP nuclear/cytoplasmic (N/C) ratio was decreased compared to healthy hiPSC-CMs, indicating a lower degree of nuclear localization and active transcription of YAP. Accordingly, DMD hiPSC-CMs exhibited lower proliferation rate (measured by Ki67 expression) compared to healthy cells, suggesting a novel target for further research and therapeutic development.

Bremner and coauthors [83] used hiPSC-CMs in the form of engineered heart tissues (EHTs) that provide a 3D physiological cell culture platform to faithfully mimic mature cardiac tissue structure [84,85,86]. Contrary to a 2D culture, comparison of DMD and healthy EHT cardiomyocytes demonstrated dysregulation of genes related to cardiac function, including many related to cardiac muscle development, action potential and contraction, as well as extracellular matrix organization in DMD cardiomyocytes. Gene Ontology (GO) analysis indicated a dysregulation of genes related to cardiac muscle development, contraction, membrane potential, intracellular Ca^2+^ handling, and extracellular matrix organization. In addition to supporting the notion of dysregulated genes in DMD cardiac pathophysiology, these results emphasize the important effect of culture structure on the maturity and gene expression patterns in hiPSC-CMs.

Marini and colleagues [87] utilized hiPSC-CMs to generate cardiac organoids (COs), 3D cellular structures possessing organotypic characteristics such as cytoarchitecture and tissue-specific physiological mechanisms [88,89,90]. Regarding changes in gene expression, Marini and coworkers found that DMD hiPSC-CMs expressed lower levels of α-actinin (*ACTN2*), pacemaker current channel *HCN4* and troponin-related genes (*TNNI1, TNNC1*, and *TNNI3*) compared to healthy cells, consistent with other reports [40,41]. DMD cardiac organoids were larger in size than healthy organoids, consistent with cellular hypertrophy, a known hallmark of cardiomyopathy [91]. Contrary to healthy COs, DMD COs lost α, β, γ, and δ-sarcoglycan expression over time, likely due to a lack of connection to dystrophin. RT-qPCR demonstrated upregulation of genes related to cardiac contractility in DMD compared to healthy Cos, including *ACTN1*, *IRX4*, *MYBPC3*, *MYL2*, *MYOM1*, *TNNC2* and *TMP1*. Marini et al. also found that ARCN1 and GORASP2, known endoplasmic reticulum (ER) stress markers, were increased in DMD cardiomyocytes compared to healthy cells, indicating a higher level of ER stress. Furthermore, immunofluorescence staining detected higher levels of NOX4, suggesting increased oxidative stress [92]. Histological examination revealed development of fibrotic-like structures in DMD COs, a finding also supported by upregulation of known fibrosis markers including *COL1A2*, *COL3A1* and *FN1*. Additionally, this group discovered increased formation of adipose tissue in DMD COs by means of H&E staining, which was validated by BODIPY staining for lipid droplets and immunostaining of PDGFRα^+^, an adipocyte marker. These findings show that DMD COs exhibit fibrotic and adipogenic characteristics, resembling clinical features of DMD cardiomyopathy [93]. Importantly, the advantages of the 3D model over 2D hiPSC-CMs are further manifested by this group’s previous report [94], which demonstrated immaturity of dystrophin-associated protein complex in 2D structures lacking protein level expression of α-, γ-, and δ-sarcoglycan. In summary, this 3D CO model enables mimicking some of the pathological findings in DMD cardiomyopathy.

## 4. Abnormal Excitation–Contraction Coupling Machinery in DMD hiPSC-CMs

Due to the important structural role of dystrophin, it is not surprising that its absence has been linked to impaired intracellular Ca^2+^ homeostasis, resulting from extracellular influx [95,96]. Additionally, elevated cellular inflammatory mediators resulting from dystrophin absence promote an increase in inducible nitric oxide synthase (iNOS), which leads to destabilization of SR ryanodine receptors (RyRs), causing SR Ca^2+^ leak into the cytosol [97,98]. Barthélémy et al. [99] further validated the key role of defective RyRs in DMD pathophysiology by demonstrating that RyR stabilizers including dantrolene, as well as Rycals S107 and ARM210, improve exon skipping efficiency in hiPSC-derived myotubes. Another possible mechanism was reported by Uchimura and Sakurai [100] who found that inhibiting store-operated Ca^2+^ channel (SOC) components STIM1 and Orai1 prevented Ca^2+^ overload and accordingly improved contractility in DMD hiPSC-derived myotubes, thus pointing to the role of these channels in excess Ca^2+^ entry. Overall, excess cytosolic Ca^2+^ can activate various proapoptotic regulators leading to cell death [101,102,103,104]. Accordingly, several studies of *mdx* mice demonstrated altered Ca^2+^ handling [105,106,107,108,109]. Guan et al. [110] were the first to demonstrate abnormal Ca^2+^ handling in hiPSC-CMs generated from DMD urine-derived stem cells (USCs), which included a lower recovery rate of Ca^2+^ transients. Additionally, early mitochondrial permeability pore opening was demonstrated in DMD hiPSC-CMs, which may be attributed to Ca^2+^ overload [111].

Additional ion channel abnormalities in DMD cells include sodium and potassium channels. *mdx* cardiomyocytes demonstrated a reduction in Na_V_1.5 channel expression and subsequently lower inward Na^+^ current [112]. In addition, a reduction in the inward rectifier K^+^ current via the Kir2.1 channel was also observed in *mdx* mice, despite unchanged channel expression levels [113]. Interestingly, Jelinkova et al. [42] found increased Kir2.1 expression but no subsequent electrophysiological changes in DMD hiPSC-CMs. *mdx* mice cardiomyocytes also displayed a decrease in K_ATP_ current, I_K,ATP_ [114,115] which is known to be associated with cardiomyopathy development [114,115]. Lastly, our group demonstrated reduced I_f_ current in DMD hiPSC-CMs despite normal HCN channel expression [40].

Contrary to enhanced I_Ca,L_ reported in *mdx* mice [106], Lin and et al. [41] found decreased I_Ca,L_ in DMD hiPSC-CMs. Using Ca^2+^ imaging, this group reported a high resting cytosolic Ca^2+^concentration. Moreover, treating DMD hiPSC-CMs with Poloxamer P188, a membrane sealant, decreased cytosolic Ca^2+^ and repressed CASP3 activation and apoptosis. These findings suggest that improving cell membrane integrity may be a beneficial strategy for prevention of cardiomyocyte loss in DMD patients.

Caluori and coauthors [116] used a system consisting of a microelectrode array (MEA) and simultaneous probing by a cantilever from an atomic force microscope (AFM) to couple electrical and mechanical recordings. In response to progressively increasing Ca^2+^ concentrations, DMD hiPSC-CMs demonstrated a lower proportional decrease in spontaneous beat rate compared to healthy CMs, which may be attributed to pre-existing Ca^2+^ stress impairing additional inward flux [117]. Introduction of verapamil, an I_Ca,L_ channel blocker, also yielded a lower proportional decrease in spontaneous beat rate compared to healthy cells.

**Figure 1:**
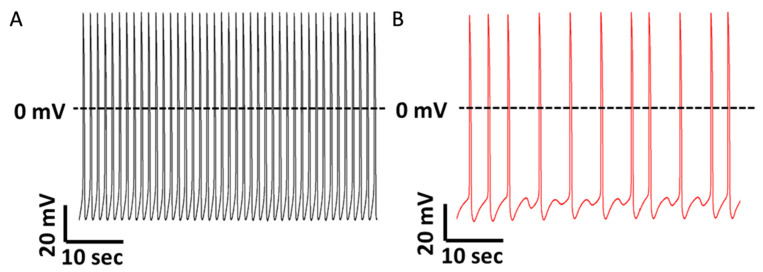
Representative spontaneous action potential recordings from healthy and DMD iPSC-CMs. (**A**) iPSC-CMs generated from a healthy donor do not manifest arrhythmias during regular spontaneous activity. (**B**) iPSC-CMs generated from a DMD patient carrying a deletion of *dystrophin* exons 8–12 display delayed afterdepolarizations (DADs) and irregular firing pattern (modified with permission from Eisen et al., 2019 [40]).

Our group [40] reported in DMD hiPSC-CMs the presence of arrhythmias represented by delayed afterdepolarizations (DADs) and irregular spontaneous firing patterns (Figure 1). DADs are usually attributed to Ca^2+^ overload [118], and this finding supports this notion. Additionally, we found increased I_Ca,L_ density consistent with previous reports in *mdx* mice [106]. Accordingly, we discovered an elevated expression of the *CACNA1C* gene, which encodes the ion channel responsible for the I_Ca,L_. Furthermore, we found a prolongation of action potential duration (APD) stemming mainly from phase 2 of the cardiac action potential, likely due to the increased I_Ca,L_ density [119]. Additionally, increased I_Ca,L_ density may be involved in the decreased response to verapamil previously reported [116].

Tsurumi et al. [120] found that diastolic and systolic Ca^2+^ concentrations measured by indo-1 fluorescence were elevated in DMD compared to healthy cardiomyocytes. Under mechanical stretching, diastolic and systolic Ca^2+^ concentrations were further increased in DMD cardiomyocytes but not in healthy cells. The elevation in intracellular Ca^2+^ concentrations, especially during diastole, correlates with diastolic dysfunction in DMD patients [121] and emphasizes the aberrant Ca^2+^ handling in DMD cardiomyocytes.

Pioner and colleagues [122] investigated mechanical and structural abnormalities in DMD hiPSC-CMs and found that they exhibited hypertrophy manifested by increased cellular length, diameter and cross-sectional area compared to healthy cardiomyocytes. Ultrastructurally, DMD hiPSC-CMs displayed sarcomeric changes including shortening of A- and Z-bands, increased percentage of identifiable I-bands, and overall greater variability in structural organization, suggesting underdeveloped sarcomere ultrastructure, consistent with previous reports of decreased transcription of sarcomeric genes [41] and reduced actin turnover [123]. These authors also demonstrated that in response to extracellular Ca^2+^ administration DMD hiPSC-CMs displayed reduced mechanical tension and prolonged relaxation. Furthermore, pCa_50_ was greater in DMD hiPSC-CMs, indicating increased Ca^2+^ sensitivity of tension generation, likely a compensatory mechanism aimed at increasing contractility in defective cells. Paced DMD hiPSC-CMs demonstrated a slower rate of contraction and relaxation compared to healthy cardiomyocytes. Post-resting period stimulation of DMD hiPSC-CMs yielded decreased twitch amplitude compared to healthy cells, suggesting a defective contractile reserve, likely due to impaired Ca^2+^ handling. Measurement of Ca^2+^ transients demonstrated low rates of rise and decay in DMD hiPSC-CMs, further supporting the notion of abnormal cellular Ca^2+^ handling. Lastly, similar to our published results [40], the spontaneous beat rate was lower in DMD compared to healthy hiPSC-CMs. Taken together, these results demonstrate the defective Ca^2+^ handling and contraction machinery in DMD hiPSC-CMs.

Kamdar and coauthors [44] tested β-adrenergic cascade characteristics in DMD hiPSC-CMs and reported an increased rate of baseline arrhythmogenic Ca^2+^ transients compared to healthy CMs. Under β-adrenergic stress introduced by isoproterenol, this group observed an increase in the frequency of arrhythmogenic Ca^2+^ traces, which were reduced by the β-adrenergic receptor blocker propranolol. Additionally, Kamdar et al. found a downregulation of genes associated with cardiac contraction and Ca^2+^ homeostasis. Overall, their results suggest an impaired β-adrenergic response in DMD cardiomyocytes, possibly correlated with autonomic dysfunction, known to affect DMD patients [124].

Jelinkova et al. [42] investigated molecular aspects of the excitation–contraction coupling (ECC) machinery and autonomic response in DMD hiPSC-CMs. Firstly, they found that compared to healthy cells, DMD hiPSCs presented lower differentiation efficiency to cardiomyocytes, manifested by a decreased fraction of spontaneously beating cells. Regarding Ca^2+^ transients, lower rates of rise and decay, as well as increased transient duration, were observed in DMD cardiomyocytes. These data support the notion that dystrophin deficiency results in ECC disturbances via Ca^2+^ handling abnormalities. They also found increased prevalence of delayed small-amplitude Ca^2+^ release events in DMD cells, which may correlate with the arrhythmogenic DADs we reported in DMD cardiomyocytes [40]. Other groups also found a weaker contraction force measured by AFM [125,126,127] in DMD cardiomyocytes. Contrary to our report, Jelinkova et al. found no difference in the spontaneous beat rate. However, similar to our findings, they found increased beat rate variability (BRV) measures in DMD cardiomyocytes compared to healthy cells [40]. DMD cardiomyocytes also displayed an abnormal response to isoproterenol and metoprolol, strengthening the notion of an abnormal β-adrenergic cascade in DMD cells. Importantly, these investigators found increased expression of β1- and β2-adrenergic receptors in DMD cardiomyocytes, likely a compensatory attempt of the defective cells.

Our group [43] investigated the β-adrenergic responsiveness and intracellular Ca^2+^ handling in DMD hiPSC-CMs. Under isoproterenol-induced β-adrenergic stimulation, DMD cardiomyocytes displayed a blunted positive inotropic response including decreased Ca^2+^ transient parameters such as amplitude, activation and relaxation rates, accompanied by corresponding changes in contraction parameters. Additionally, DMD cardiomyocytes did not exhibit a depressed chronotropic response to isoproterenol, suggesting the mechanism underlying the blunted inotropic response is not an impaired β-adrenergic cascade. In response to increasing extracellular Ca^2+^ concentration, known to induce augmented SR Ca^2+^ release and positive inotropy, DMD cells displayed smaller Ca^2+^ and contraction parameters compared to healthy cardiomyocytes. Lastly, we tested the effect of caffeine, which induces SR Ca^2+^ release, the common pathway of β-adrenergic stimulation and Ca^2+^ administration inducing positive inotropy. DMD cardiomyocytes displayed a blunted response to caffeine, including smaller Ca^2+^ amplitude and a shorter recovery time compared to healthy cells. In support of the notion of depleted SR Ca^2+^ stores in DMD cardiomyocytes, administration of ryanodine and cyclopiazonic acid (CPA), a SERCA inhibitor, resulted in a smaller inhibitory effect compared to healthy cells represented by a smaller relative decrease in Ca^2+^ amplitude. These results, combined with the reported changes in expression of genes related to Ca^2+^ handling, SERCA2, ADRβ1 and AC, point to abnormal Ca^2+^ handling as a key factor underlying abnormal β-adrenergic response in DMD.

YAP activity is known to increase due to mechano-transduction converting physical to electrochemical stimulus, regulated by actin dynamics [128,129]. Therefore, Yasutake et al. [81] assessed the actin state to determine its possible involvement in altered YAP activity in DMD hiPSC-CMs. Indeed, DMD cardiomyocytes were found to be initially smaller and rounder than healthy CMs and had a lower number of nuclei, in addition to failing to morphologically change with maturation as healthy cardiomyocytes, supporting the notion that DMD cardiomyocytes fail to progress morphologically in a proper manner. Immunofluorescence staining demonstrated a disrupted actin structure in DMD hiPSC-CMs as well, indicating a possible link between abnormal actin and YAP activity in DMD cardiomyocytes.

Chang and colleagues [77] devised a unique bioengineered traction force microscopy platform which enables mimicking stiffness of fibrotic heart tissue, a key feature of DMD cardiomyopathy, as well as controlling cardiomyocyte orientation. DMD hiPSC-CMs displayed increased resting [Ca^2+^], decreased Ca^2+^ transient amplitude, increased transient duration and increased transient decay rate, measured by Fura 2 fluorescence. These results demonstrate that impaired Ca^2+^ handling is involved in DMD pathophysiology at the tissue level.

Bremner and coauthors [83] measured contractile force in electrically stimulated hiPSC-CMs 3D EHT to mimic mature cardiac tissue structure [84,85,86]. DMD cardiomyocytes generated decreased twitch force and contraction kinetic parameters compared to healthy cardiomyocytes, implying lower contractile performance. Staining of Z-disc and α-actinin followed by confocal imaging revealed that DMD cardiomyocytes had shorter sarcomere length compared to healthy cardiomyocytes, likely contributing to the lower contractile force. Using Fura-2 fluorescence, it was found that DMD cardiomyocytes displayed a higher baseline of cytosolic [Ca^2+^] and increased transient peak, but overall decreased amplitude compared to healthy cells. DMD cells also exhibited slower rates of rise and decay of Ca^2+^ transients. These results emphasize once more abnormal Ca^2+^ handling as a key pathophysiological feature of DMD. Consistent with our previous report [40], DMD EHTs displayed increased BRV compared to healthy cells, strengthening the 3D structure as a valid important model for investigating DMD cardiomyopathy.

Pioner et al. [130] investigated in DMD hiPSC-CMs the maturation of the Ca^2+^ handling machinery and adaptation to changes in substrate stiffness. Analysis of Ca^2+^ transients revealed that DMD cardiomyocytes exhibited an initial smaller Ca^2+^ transient amplitude and a smaller increase in the transient amplitude with maturation compared to healthy cardiomyocytes. Additionally, post-rest contraction potentiation was lower in DMD compared to healthy cardiomyocytes, indicating lower SR calcium storage and release. Ca^2+^/calmodulin-dependent protein kinase II (CaMKII) levels were higher in DMD compared to healthy cardiomyocytes; consequently, the RyR2 CaMKII phosphorylation site S2814, was more phosphorylated. This increased phosphorylation may have accounted for the SR Ca^2+^ leak, resulting in the reduced Ca^2+^ transient amplitude in DMD cardiomyocytes. Compared to healthy cells, DMD hiPSC-CMs exhibited reduced Ca^2+^ transient amplitude on both soft and stiff substrates. Furthermore, while healthy cardiomyocytes displayed an accelerated decay of Ca^2+^ transient amplitude when cultured on a stiffer substrate, DMD cells did not differ in their kinetics between the substrates, indicating failure to adapt normally to differences in tissue stiffness. These findings demonstrate the abnormal Ca^2+^ handling of DMD cardiomyocytes and specifically the inability to adapt their Ca^2+^ homeostasis in response to changes in ECM stiffness, which may be a key factor of the DMD cardiac pathophysiology.

## 5. Alterations in Cellular Energy and Metabolism in DMD hiPSC-CMs

Emerging evidence from recent years suggests that metabolic and mitochondrial dysfunction play prominent roles in DMD pathophysiology [131,132,133,134,135,136,137]. Afzal et al. [138] tested the effects of nicorandil, a known NO donor, K_ATP_ channel opener and an antioxidant [139,140,141] on DMD hiPSC-CMs. The group found that in DMD hiPSC-CMs, nicorandil reduced H_2_O_2_-induced stress related LDH release and cell death to levels similar to healthy cells, through a mechanism involving the NO–cGMP pathway and K_ATP_ channel opening. Nicorandil also decreased ROS levels and maintained mitochondrial membrane integrity following oxidative stress, an effect mediated via increase of SOD2 expression which converts superoxide to hydrogen peroxide. Lastly, nicorandil was also shown to decrease cytosolic hydrogen peroxide production in DMD hiPSC-CMs. Although further research is required, these findings provide evidence that nicorandil may have a potential to protect against stress-induced cardiac involvement in DMD.

Gartz and colleagues [142] investigated cardioprotective effects of exosomes on DMD cardiomyocytes. They found that exosomes collected from conditioned media of both DMD and healthy hiPSC-CMs decreased injury-induced ROS levels in DMD cells. Additionally, exosomes led to inhibition of Bax expression and mitochondrial translocation, which are known to trigger opening of the mitochondrial permeability transition pore (mPTP), resulting in caspase activation and cell death [143]. Furthermore, exosomes were found to reduce stress-induced loss of mitochondrial membrane integrity and early mPTP opening time in DMD cardiomyocytes, as well as decreasing caspase 3/7 levels and subsequent apoptosis. By pretreating exosomes with trypsin, the researchers discovered that surface proteins are required to exert the protective effect. Treated exosomes failed to initiate ERK1/2 phosphorylation, an important component of the mitogen-activated protein kinase (MAPK)-mediated response which is responsible for dictating cell death or survival [144]. Additionally, inhibition of p38 MAPK reversed exosome-protective effects, indicating its specific involvement in cardio-protection. These results demonstrate the protective potential of cardiomyocyte-secreted exosomes in DMD cardiomyocytes, specifically the involvement of surface proteins ERK1/2 and p38 MAPK. Gartz and coauthors [145] further investigated the involvement of secreted exosomes in the pathological response of DMD hiPSC-CMs to metabolic stress. Exosomes, known to play an important role in intercellular communication and signaling [142,146,147,148], were studied in DMD [146,147,148,149,150] and found to be involved in modulation of inflammatory processes, oxidative injury, mitochondrial respiration, myocyte differentiation and cell death. In this study, the researchers identified several exosomal micro RNAs (exomiRs) dysregulated in DMD. Specifically, mir-339-5p was found to be upregulated in DMD exosomes and DMD hiPSC-CMs. The mir-339-5p level was also found to increase with cardiac injury and age in *mdx* mice, rendering it a potential cardiomyopathy-sensitive target in DMD. Interestingly, in DMD hiPSC-CMs, miR-339-5p-containing exosomes were found to be involved in stress-related mitochondrial-dependent cell death. Specifically, miR-339-5p, known to directly bind to MDM2, a p53 regulator leading to its downregulation [151], was also found to decrease MDM2 levels in DMD hiPSC-CMs. MDM2 was then reported to be downregulated in stressed DMD hiPSC-CMs. Lastly, miR-339-5p knockdown in DMD cardiomyocytes led to the preservation of mitochondrial membrane potential and reduction in stress-induced cell death, indicating its important role in modulating the cellular response to stress in DMD hiPSC-CMs. Collectively, these findings emphasize the importance of exosomal miR-339-5p involvement in cellular stress response, which may also serve as a basis for future research as a therapeutic target.

Duelen and coauthors [152] investigated the involvement of NOX4 in oxidative stress in DMD hiPSC-CMs. NOX enzymes, known to generate reactive oxygen species (ROS) in various cellular processes [153], are also involved in cardiovascular diseases [154,155]. On the basis of this knowledge, these researchers targeted NOX4 (mainly expressed in cardiomyocytes) in DMD hiPSC-CMs. Contrary to our report and that in *mdx* mice [40,106], DMD cardiomyocytes displayed a reduction in I_Ca,L_ density, although, similarly to our findings, they observed arrhythmogenic firing patterns including DADs and oscillatory prepotentials (OPPs). Another finding in line with ours was APD prolongation in DMD cells. Cell death, measured by flow cytometric analyses using annexin V and 7-amino-actinomycin D (7AAD), revealed that DMD cardiomyocytes underwent accelerated cell death compared to healthy cells. Furthermore, DMD cells had a higher ROS content compared to healthy cardiomyocytes. Treatment with N-acetyl-L-cysteine (NAC), ataluren (PTC124) and idebenone increased DMD cell survival and reduced ROS levels. By means of JC-1, a fluorescent voltage-sensitive dye with membrane-permeant fluorescent lipophilic cationic properties, Lin and coworkers measured mitochondrial membrane potential. Similar to Lin et al. [41], DMD hiPSC-CMs displayed increased levels of mitochondrial depolarization, which was improved by treatment with NAC, ataluren, and idebenone. Consistent with previous reports of increased NOX4 expression in heart failure [154,155,156,157], NOX4 was also upregulated in DMD hiPSC-CMs. Treatment with PTC124, NAC and idebenone reduced NOX4 expression. To assess the role of NOX4 in increased ROS levels, antisense locked nucleic acid (LNA) GapmeRs was used to degrade NOX4 mRNA. Indeed, NOX4 degradation led to reduced NADPH-dependent ROS production. DMD cardiomyocytes displayed increased NOX4 levels, and following addition of idebenone, ROS production was reduced. Furthermore, addition of ATP, a NOX4 regulator, and idebenone treatment also reduced ROS production. Lastly, contractile function assessed in 3D EHT constructs showed that DMD cardiomyocytes displayed lower contraction amplitude compared to healthy cells; this decrease was rescued by PTC124, NAC, and idebenone.

Our group investigated various metabolic and bioenergetic aberrations in DMD cardiomyocytes [158]. We first focused on polar metabolites (such as phospho-sugars, organic acids, nucleotides, and fatty acids) by means of liquid chromatography/mass spectrometry (LC–MS)-based metabolomics to study the central carbon metabolism of DMD cardiomyocytes. Indeed, nine metabolites differed in DMD cardiomyocytes compared to healthy cells, pointing primarily toward impairment in fatty acid oxidation. Next, we used labeled glucose tracing to study cellular glucose-derived metabolites. Indeed, DMD cardiomyocytes exhibited lower levels of glucose-derived carbon into the TCA cycle, indicating decreased mitochondrial glucose oxidation. Seahorse XFe96 flux analyzer was used to assess mitochondrial respiration and mitochondrial oxidative phosphorylation by measuring the oxygen consumption rate (OCR). DMD cardiomyocytes displayed decreased basal respiration and ATP production coupled to oxygen consumption, compared to healthy CMs. Interestingly, uncoupling ATP synthesis from the mitochondrial respiratory chain restored respiration rate, suggesting defective mitochondrial ATP synthase (Complex V). To examine mitochondrial number and morphology, electron microscopy (EM) analysis was performed. DMD cells contained a higher number of mitochondria compared to healthy CMs. Additionally, a higher frequency of aberrations, including increased size, disrupted cristae and multiple focal swelling areas, were seen in DMD cardiomyocytes. To further investigate the association between morphological abnormalities and impaired mitochondrial function, we measured mitochondrial activity by means of live confocal 3D imaging. Florescent staining revealed that mitochondrial activity was attenuated compared to healthy cells. Taken together, these findings suggest that bioenergetic and metabolic impairments are involved in DMD cardiac pathophysiology and should be further investigated in search of novel therapeutic targets.

## 6. Gene Therapy and Gene Editing in DMD hiPSC-CMs

Current treatments for DMD can at best slow the disease course and alleviate some symptoms [19,159,160]. However, these treatments do not fundamentally change the outcome, and patients rarely live beyond their late 20 s [18,161]. Furthermore, since around one-third of DMD cases originate from *de novo* mutations with no familial background [162,163], preconception genetic testing is expected to miss a significant proportion of cases. Hence, to truly cure DMD, genetic correction is needed.

**Figure 2:**
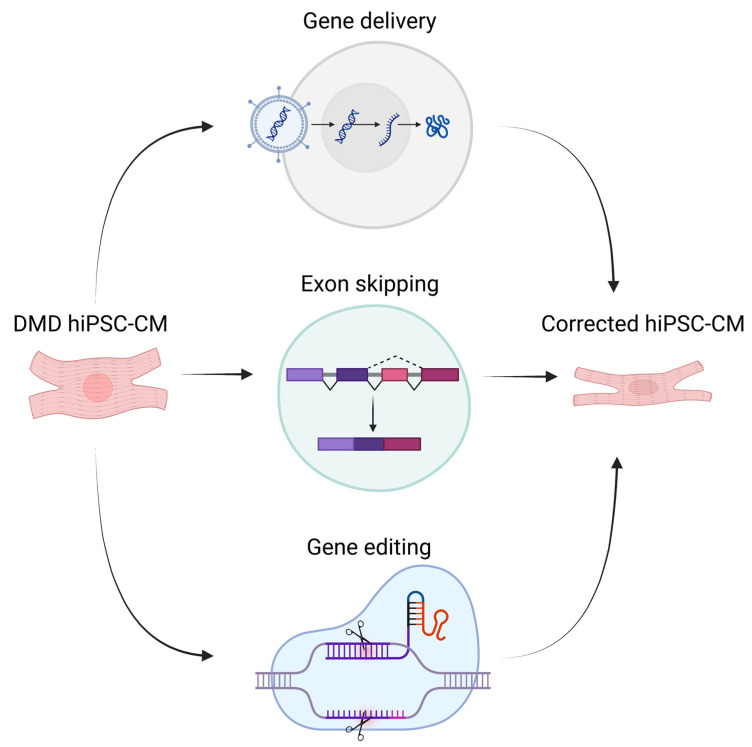
Illustration of different genetic therapeutic methods for DMD (created with BioRender.com).

Different methods can be used to attempt genetic repair (Figure 2). Early efforts to genetically correct DMD hiPSCs were conducted using gene therapy. Kazuki et al. [164] demonstrated the successful delivery of a human artificial chromosome (HAC) containing the complete *dystrophin* gene. Zatti and coauthors [165] successfully delivered an HAC containing WT *dystrophin* into DMD hiPSC-CMs; these authors found that differentiated cardiomyocytes expressed all dystrophin isoforms and displayed corrected protein localization, in addition to normal Ca^2+^ transients. Choi and colleagues [166] also reported correction of abnormal *dystrophin* gene expression profiles upon HAC delivery into DMD cardiomyocytes. Dick and coauthors [167] chose minigene delivery and exon-skipping to restore *dystrophin* expression in DMD cardiomyocytes. Additionally, Howard et al. [168] found that micro-dystrophin transgene containing the main functional domains was sufficient to preserve cardiac function and prevent fibrosis and inflammation in a DMD mouse model. While these techniques are useful tools for research, clinical application is not easily feasible as delivered HACs and minigenes are only transiently expressed in host cells [169]. In addition, delivery of such large complexes is a difficult challenge and carries a risk of nonspecific integration to the host DNA [170,171].

Another approach to correct DMD mutations is by editing the mutated gene directly. Gene editing requires a break in the DNA sequence at a specific position, followed by repair, which includes replacement of excised DNA with a new sequence. Cellular DNA repair mechanisms include two options [172,173,174]. The first is homology-directed repair (HDR), which is based on a new sequence introduced to repair double-strand breaks (DSBs). This enables precise correction of mutations in the DNA sequence. The DNA template used for repair can be coupled and delivered together with an endonuclease which performs the DSBs. Delivering a template to be utilized by HDR is the preferred mechanism for correction of small genetic mutations which require an insertion of a relatively small new sequence [175]. The second option is nonhomologous end-joining (NHEJ), which joins two DNA break ends, but at the cost of frequent small insertions/deletions (indels), potentially disrupting the open reading frame (ORF) and subsequently leading to RNA degradation through the nonsense-mediated decay (NMD) mechanism or production of a nonfunctional protein. NHEJ is useful to correct large frame-shifting deletions or duplications, by means of deleting additional base pairs to achieve ORF restoration or excising the duplicated segment to accomplish complete correction, respectively [175,176].

To achieve safeness and effectiveness, the process of introducing an exogenous endonuclease alongside a DNA template must be highly precise with minimal off-target effects. To date, three main systems are in use for preforming DNA DSBs and editing:

- Clustered regularly interspaced short palindromic repeats (CRISPR)/CRISPR-associated protein 9 (Cas9) endonuclease comprises an anti-bacteriophage bacterial system [177]. CRISPR are bacterial DNA segments used to store antibacterial genetic information. Upon infection with a virus containing RNA homologous to RNA transcribed from the CRISPR, effector proteins can be guided by the latter to cleave the virus. Cleavage is performed by endonucleases such as Cas9, Cpf1, and Cas12. The guiding RNA sequence (gRNA) can be edited and, together with the endonuclease, delivered into cells using different types of vectors [178,179].
- Zinc finger nucleases (ZFNs) are a group of enzymes capable of cleaving specific sites in DNA. ZFNs consist of DNA-binding zinc finger arrays and a FokI-based endonuclease [180]. Despite high specificity of ZFN binding, the process of producing precise zinc fingers for specific DNA segments is considered relatively difficult and expensive [181].
- Transcription activator-like effector nucleases (TALENs) are based on proteins produced by *Xanthomonas* bacteria which are capable of plant gene expression alteration [182,183]. Like ZFNs, TALENs production is difficult and expensive compared to CRISPR and its associated endonucleases [184,185].

Off-target effects are a major concern when considering the clinical application of gene editing. These include possible disruption of oncogenes, tumor suppression genes and DNA repair genes [176,186,187]. An additional issue is the possibility of an immune response against elements of the delivered DNA-binding and endonuclease complex, or even against corrected gene products [176,188]. Despite these concerns, it is widely agreed among researchers and clinicians that genome editing carries the potential to cure genetic diseases previously considered incurable.

To develop an approach which potentially benefits a larger target population, Young et al. [189] utilized the CRISPR/Cas9 system to delete exons 45–55 and most of their adjacent introns. The rationale is that, since most DMD patients suffer from out-of-frame mutations located within the “hotspot” of exons 45–55, deletion of these exons and restoration of the ORF will potentially benefit most of the patients [190,191]. Moreover, in-frame mutations in the same region are associated with the milder dystropathy, Becker muscular dystrophy (BMD) [192]. The successful restoration of the reading frame was confirmed via sequencing, and the corresponding protein expression was validated by immunohistochemistry and Western blot (WB). As a functional test, hiPSC-CMs and skeletal myocytes were exposed to osmotic stress. Indeed, in hypotonic solution, creatine kinase (CK) release levels were reduced in corrected cells to levels similar to healthy cardiomyocytes, indicating that the restored *dystrophin* provided sufficient membrane stability. Furthermore, miR-31 levels, which were reportedly high in DMD cells [193,194], were also reduced to levels comparable to BMD cells. Lastly, immunostaining and WB demonstrated successful restoration of β-dystroglycan in corrected skeletal myocytes. Additionally, hiPSC-derived skeletal myocytes were injected into NOD-*scid* IL2Rgamma (NSG) *mdx* mice, and dystrophin and β-dystroglycan were found only in the corrected and healthy cardiomyocytes. In conclusion, ORF restoration reinstated key functional cellular aspects of dystrophin, and this approach was proven as an important therapeutic strategy.

Zhang and colleagues [195] applied a similar principle, as skipping exon 51 can potentially restore the ORF in ~13% of DMD exon deletion mutations [196]. This group used two Cpf1-mediated CRISPR approaches to restore the ORF in hiPSCs carrying an out-of-frame deletion of exons 48–50. The first approach involved a single gRNA aimed at introducing indels in exon 51 by nonhomologous end joining (NHEJ). Indeed, corrected hiPSCs were reframed successfully and displayed an in-frame connection of exons 47 and 51. Another approach involved two gRNAs to achieve ORF restoration by skipping exon 51 and connecting exon 47 and 52. Restoration of the truncated protein was confirmed by WB analysis and immunocytochemistry. As a functional test, the mitochondrial DNA (mtDNA) and cellular respiration rate of hiPSC-CMs were measured. Corrected cells displayed increased mitochondrial numbers and oxygen consumption rate (OCR) compared to DMD cells and comparable to WT. Overall, these results demonstrate another promising approach in which individual DMD patients with different mutations may benefit from a “generic” ORF restoration.

Kyrychenko and coauthors [197] compared three approaches to restore the ORF in hiPSCs carrying a DMD mutation (deletion of exons 8–9) involving the actin-binding domain-1 (ABD-1) [12]. The first approach was deleting exons 3–7, leaving out actin-binding sites 1 and 2 (ABS-1 and ABS-2). Another strategy involved deleting exons 6–7 and excising ABS-3. The third method was deleting exons 7–11, leaving all three ABS regions intact. The respective dystrophin expression was validated by WB analysis and immunocytochemistry. As a functional assessment, spontaneous Ca^2+^ transients were analyzed in hiPSC-CMs. Indeed, release and reuptake parameters, including time to peak, Ca^2+^ decay rate, and transient duration, were higher in the uncorrected DMD hiPSC-CMs compared to WT and isogenic cells. Interestingly, while corrected cells with exons 3–9 deleted displayed normalized Ca^2+^ transient properties, corrected hiPSC-CMs carrying a deletion of exons 6–9 or 7–11 displayed only partial improvement of the Ca^2+^ transient parameters. These results indicate that, among the three approaches of ORF restoration, the one leading to a deletion of exons 3–9 yields the most functioning protein. Additional functional assessment was performed by means of engineered heart muscle (EHM). Consistent with the Ca^2+^ transient parameters results, enhanced contractile performance was observed in all corrected cell lines, with the most prominent effect in hiPSC-CMs carrying the deletion of exons 3–9. Overall, these results suggest that, of the three approaches, the correction leading to a deletion of exons 3–9 was the most effective in restoring functionality, implying the importance of ABS-3 over ABS-1 and ABS-2 of the ABD-1 domain. This also demonstrates the importance of functional testing, as they serve as a measure to evaluate the different approaches, specifically because there is no conclusive explanation as to why the correction resulting in the three ABS regions being intact (and a deletion of exons 7–11) was less effective than the approach leaving out ABS-1 and ABS-2.

Long et al. [198] identified single gRNAs capable of skipping and restoring the ORF in ~60% of DMD mutations together with the CRISPR/Cas9 system [199]. As a demonstration, they treated three different types of mutations. DMD hiPSCs carrying a deletion of exons 48–50 were corrected by destruction of the splice acceptor in exon 51, thereby allowing splicing of exon 47 to exon 52 and restoration of the ORF. DMD cells carrying a nonsense point mutation in intron 47 were corrected directly leading to permanent skipping of the pseudo-exon and restoring full-length dystrophin. Lastly, DMD hiPSCs carrying a duplication mutation of exons 55–59 were corrected by targeting the junction of intron 54 and exon 55, thus leaving out the duplicated region. Correction was confirmed via RT-PCR, and dystrophin levels were measured by immunocytochemistry and WB. Three-dimensional engineered heart muscle (3D-EHM) was used to assess functionality of WT, DMD, and corrected hiPSC-CMs; the force of contraction (FOC) was improved in corrected cells. Furthermore, this group found that 30–50% of cardiomyocytes need to be repaired for partial (30%) or maximal (50%) rescue of the contractile phenotype. In summary, these results support the notion of developing treatments benefiting groups of patients rather than individual mutations. Furthermore, their insistence on using single gRNAs simplifies the process and potentially increases efficacy.

Yuan and coauthors [200] utilized the previously published method of fusing nuclease-inactivated Cas9 with an activation-induced cytidine deaminase (AID) to convert targeted *C* to *T* or *G* to *A*, and subsequently restore the ORF in DMD hiPSCs lacking exon 51 [201,202]. The group successfully skipped exon 50 of *dystrophin*, thus restoring the ORF in 99.9% of *dystrophin* transcripts, as demonstrated by high-throughput sequencing (HTS). Corrected hiPSC-CMs released normal levels of creatine kinase (CK). Additionally, expression of miR-31 was suppressed in the corrected hiPSC-CMs, and β-dystroglycan levels were increased to levels comparable with healthy hiPSC-CMs. Importantly, contrary to CRISPR/Cas9 approaches which involve DSBs, targeted AID-mediated mutagenesis (TAM) does not require NHEJ or HDR; therefore, it is significantly less prone to indels, with higher correction efficiency [203]. These benefits strongly suggest that TAM is superior to other CRISPR/Cas9 correction methods for DMD and should be favored when applicable.

Sato and colleagues [204] investigated the effects of exon skipping on Ca^2+^ handling in DMD hiPSC-CMs. They used phosphorodiamidate morpholino oligomers (PMOs) to skip exon 45 and restore the ORF in cells carrying a deletion of *dystrophin* exons 46–55. By means of Fluo-4 they measured Ca^2+^ transient parameters following treatment and detected increased amplitude, as well as a decrease in the rates of rise and decay, all improvements resembling healthy cells. Additionally, the number of fluorescent-detected irregular pattern events suggestive of arrhythmogenicity decreased following exon 45 skipping. Taken together, these results demonstrate the effectiveness of the exon-skipping strategy to improve functional abnormalities in DMD cardiomyocytes.

Chemello et al. [205] investigated correction of exon deletion mutations using base and prime editing. For base editing, they used an adenine base editor approach with ABEmax-SpCas9 to induce exon 50 skipping by means of swapping the *GT* to *GC* sequence and restoring the ORF in DMD cells carrying an exon 51 deletion. The prime-editing strategy was used to introduce a swap of the *TT* to *GT* sequence in exon 52, resulting in ORF restoration. RT-PCR, sequencing, WB analysis, and immunocytochemistry confirmed correction and the resulting truncated protein. Next, the researchers tested the effect of correction on cardiomyocytes treated with isoproterenol. Indeed, corrected DMD cardiomyocytes displayed a reduction in arrhythmogenic Ca^2+^ transients compared to DMD cardiomyocytes, similar to healthy cardiomyocytes. Overall, these results demonstrate the feasibility of both genetic correction approaches in alleviating DMD cardiac pathophysiology.

Zhang and coauthors [206] devised a new approach for delivering Cas9 as part of CRISPR/Cas9 genome editing. Instead of using *Streptococcus pyogenes* Cas9 (SpCas9), which requires a dedicated adeno-associated virus (AAV) vector aside from the sgRNA vector, they used a Cas9 ortholog from *Staphylococcus aureus* (SaCas9), which is small enough to be packed together with sgRNA into a single AAV vector. This strategy was then utilized to deliver SaCas9-mediated single-cut gene editing to correct an exon 48–50 DMD deletion, avoiding possible unwanted genomic modifications due to the “double-cut” strategy with dual sgRNAs [207,208]. Their aim was restoration of the ORF of exon 51 via a two-nucleotide deletion. Indeed, the reading frame of hiPSCs carrying a DMD deletion of exons 48–50 was corrected accordingly. Furthermore, following correction, Ca^2+^ transient parameters in hiPSC-CMs, such as time to peak and rate of decay, were comparable to healthy cells, contrary to their abnormal elevation prior to correction. Additionally, genotoxicity analysis demonstrated no significant off-target editing, suggesting that this method is safe and effective at restoring the ORF and achieving functional restoration in DMD hiPSC-CMs.

Atmanli and colleagues [209] investigated various effects of CRISPR/Cas9-mediated corrections in DMD hiPSC-CMs. They generated two different hiPSC lines to restore the ORF in cells carrying a deletion of *DMD* exon 44: one using a single-nucleotide insertion to achieve reframing and the other by skipping exon 45 to restore the ORF. The correction of DMD hiPSC-CMs restored cellular area, volume and sarcomere length back to normal. DMD cardiomyocytes showed Ca^2+^ handling abnormalities including increased release and reuptake times, in addition to impaired localization of RYR2s, all of which were corrected with the truncated *dystrophin* lines. To test the effects of the corrections on contractility, the researchers used a polydimethylsiloxane matrix as a substrate for seeding cardiomyocytes, allowing auxotonic contraction. By means of video detection they demonstrated in DMD cells decreased systolic force and prolonged relaxation time, which were corrected in the truncated *DMD* lines. Furthermore, they found that ORF restoration reversed changes in gene expression, including two hallmark DCM transcripts, NPPA and NPPB [210], which were downregulated in DMD compared to corrected hiPSC-CMs. The researchers also tested changes in membrane tension by means of fluorescent membrane tension probe Flipper-TR [211]. These researchers found that DMD cardiomyocytes displayed higher membrane tension compared to healthy and corrected cells, demonstrating the important structural role of dystrophin, which is also preserved with the truncated protein. Lastly, the investigators corrected DMD cardiomyocytes directly, after the appearance of cellular abnormalities. Interestingly, after loading cardiomyocytes with the voltage-sensitive probe FluoVolt to measure arrhythmogenicity, they found a reduction in arrhythmogenicity in corrected and healthy cells compared to DMD cardiomyocytes. In summary, this robust work demonstrated the important functional, transcriptional and structural abnormalities in DMD cardiomyocytes, which were all rescued with CRISPR/Cas9-mediated ORF restoration.

## 7. Summary

This article reviewed the DMD cardiomyopathy-related research conducted in recent years in hiPSCs-CMs, as summarized in Table 1. The importance of investigating DMD pathophysiology in an in vitro human model is emphasized by the limitations of the current popular animal model, the *mdx* mouse [26,27,28,212]. Furthermore, the breakthrough reprograming technique enables the generation of patient- and mutation-specific hiPSC lines, providing an endless pool of cells carrying specific mutations for research [49,50,51,55,56]. Indeed, research on DMD hiPSC-CMs has yielded novel findings thus far, as well as validation of results previously reported in other models. Furthermore, with the introduction of gene-editing techniques, promising therapeutic possibilities are becoming more likely, including complete genetic cure. These need to be tested first in vitro, and hiPSCs are the ultimate model for such research. Furthermore, gene editing enables the generation of isogenic controls which provide significantly stronger validation of results obtained using the hiPSC model. Lastly, although extensive research is needed before clinical application, hiPSCs and hiPSC-derived cells may be used in the future in regenerative medicine to repair damaged tissue which may otherwise not be treatable.

## Figures and Tables

**Table 1:** Summary of DMD hiPSC-CM research papers in recent years.

Reference	Model Type (2D/3D)	Cell Origin (Patient-Derived/Edited Healthy Donor-Derived)	Main Findings
Dick et al., 2013 [167]	3D (EBs)	Patient-derived	Dystrophin restoration via minigene delivery and exon skipping
Guan et al., 2014 [110]	2D	Patient-derived	Ca^2+^ abnormalities, mitochondria damage
Zatti et al., 2014 [165]	3D (EBs)	Patient-derived	Dystrophin restoration via human artificial chromosome delivery and subsequent correction of Ca^2+^ abnormalities
Lin et al., 2015 [41]	3D (EBs)	Patient-derived	Ca^2+^ abnormalities, mitochondria damage
Afzal et al., 2016 [138]	2D	Patient-derived	Nicorandil protection against stress-induced cardiomyocyte death
Young et al., 2016 [189]	2D	Patient-derived	CRISPR/Cas9-mediated open reading frame restoration
Kyrychenko et al., 2017 [197]	2D	Edited healthy donor-derived	Comparison of different CRISPR/Cas9-mediated open reading frame restoration targets
Zhang et al., 2017 [195]	2D	Patient-derived	CRISPR/Cpf1-mediated open reading frame restoration
Chang et al., 2018 [72]	2D	Patient-derived	Telomere shortening in DMD cardiomyopathy
Gartz et al., 2018 [142]	2D	Patient-derived, edited healthy donor-derived	Exosome-mediated cardioprotection in DMD CMs
Long et al., 2018 [198]	3D (EHM)	Patient-derived	CRISPR/Cas9-mediated open reading frame restoration in EHM
Yuan et al., 2018 [200]	2D	Patient-derived	CRISPR-guided cytidine deaminase-mediated open reading frame restoration
Caluori et al., 2019 [116]	3D (EBs)	Patient-derived	Electrophysiological and mechanical abnormalities
Eisen et al., 2019 [40]	2D	Patient-derived	Electrophysiological abnormalities
Farini et al., 2019 [79]	2D	Patient-derived	Immunoproteasome dysregulation
Sato et al., 2019 [204]	2D	Patient-derived	Exon skipping-mediated open reading frame restoration
Tsurumi et al., 2019 [120]	2D	Patient-derived	Ca^2+^ abnormalities
Jelinkova et al., 2020 [42]	3D (EBs)	Patient-derived, edited healthy donor-derived	Electrophysiological and Ca^2+^ abnormalities, impaired β-adrenergic cascade
Kamdar et al., 2020 [44]	2D	Patient-derived	Impaired β-adrenergic cascade
Pioner et al., 2020 [122]	2D	Patient-derived, edited healthy donor-derived	Mechanical and Ca^2+^ abnormalities
Atmanli et al., 2021 [209]	2D	Patient-derived	Functional, transcriptional, and structural changes following CRISPR/Cas9-mediated open reading frame restoration
Chang et al., 2021 [77]	2D	Patient-derived	Mechanical and Ca^2+^ abnormalities, telomere shortening
Chemello et al., 2021 [205]	2D	Edited healthy donor-derived	Base and prime editing-mediated open reading frame restoration
Gartz et al., 2021 [145]	2D	Patient-derived, edited healthy donor-derived	Exosome-mediated pathology in DMD CMs
Mekies et al., 2021 [43]	2D	Patient-derived	Ca^2+^ abnormalities, impaired β-adrenergic cascade
Yasutake et al., 2021 [81]	2D	Patient-derived	Decreased yes-associated protein activity
Zhang et al., 2021 [206]	2D	Patient-derived	Usage of *Staphylococcus aureus* Cas9 (SaCas9) for single cut-mediated open reading frame restoration
Bremner et al., 2022 [83]	3D (EHT)	Edited healthy donor-derived	Mechanical and Ca^2+^ abnormalities in EHT
Duelen et al., 2022 [152]	3D (EHT)	Patient-derived	Role of NADPH oxidase 4 in DMD cardiomyopathy-related oxidative stress
Marini et al., 2022 [87]	3D (COs)	Patient-derived	Morphological and gene expression changes in COs
Pioner et al., 2022 [130]	2D	Patient-derived, edited healthy donor-derived	Mechanical and Ca^2+^ abnormalities
Willi et al., 2022 [158]	2D	Patient-derived	Bioenergetic and metabolic abnormalities

COs—cardiac organoids, EBs—embryoid bodies, EHM—engineered heart muscle, EHT—engineered heart tissue.

## Data Availability

Not applicable.
